# Puerarin attenuates *S. aureus*-induced mastitis through inhibiting inflammation and ferroptosis by activating the SIRT1/p53/SLC7A11 signaling pathway

**DOI:** 10.1128/spectrum.02859-24

**Published:** 2025-07-22

**Authors:** Yang Yang, Liang Sun, Changlong Song, Di Zhou

**Affiliations:** 1 Department of Oncology and Hematology, China-Japan Union Hospital of Jilin University 74569, Changchun, Jilin, China; 2 Department of Breast Surgery, China-Japan Union Hospital of Jilin University 74569, Changchun, Jilin, China; Icahn School of Medicine at Mount Sinai, New York, New York, USA

**Keywords:** mastitis, ferroptosis, puerarin, inflammation, SIRT1

## Abstract

Mastitis, one of the most prevalent diseases in humans and animals, is commonly caused by local infection of the mammary gland. Puerarin inhibits mitochondrial oxidative stress and acute liver injury in mice. However, the therapeutic effect of puerarin on *Staphylococcus aureus*-induced mastitis is still not well understood. This study was designed to investigate the therapeutic role and mechanism of puerarin on *S. aureus*-induced mastitis. *S. aureus* was injected through the mammary gland to induce mouse mastitis. Puerarin was administered to mastitis mice via intraperitoneal injection. The iron concentration was measured using an iron assay. TNF-α and IL-1β levels in the mammary gland tissues were assessed using the ELISA kits. The signaling pathways were detected by western blot analysis. Hematoxylin and eosin staining revealed that puerarin reversed pathological injury to mammary gland tissue induced by *S. aureus*. Puerarin also inhibited TNF-α and IL-1β production induced by *S. aureus*. The significant increases in iron concentration, myeloperoxidase activity, and the malondialdehyde (MDA) level in the mammary gland tissue were attenuated by puerarin. Moreover, puerarin treatment significantly reversed the decreases in the relative glutathione (GSH) and GPX4 expression. In addition, puerarin increased the expression of SIRT1 and solute carrier family 7 member 11 (SLC7A11) and decreased p53 expression. *In vitro*, the inhibitory effects of puerarin on *S. aureus*-induced inflammation and ferroptosis were prevented by the SIRT1 inhibitor EX-527. In conclusion, the therapeutic mechanism of puerarin involves the activation of the SIRT1/p53/SLC7A11 pathway to inhibit ferroptosis and the inflammatory response.

**IMPORTANCE:**

Antibiotics are the main drugs used to treat mastitis, but they can easily cause bacterial resistance and food safety issues. Therefore, finding safe and effective drugs is crucial to prevent and treat mastitis. Puerarin is the main isoflavonoid active ingredient in *Pueraria lobata* and has a wide range of pharmacological effects. We found that it could attenuate mastitis induced by *Staphylococcus aureus*.

## INTRODUCTION

Mastitis is a very common disease in humans and is usually caused by local infection through the mammary gland (1). Researchers generally believe that the leading cause of mastitis is through pathogens such as *Staphylococcus aureus (S. aureus*), *Streptococcus pneumoniae*, and *Escherichia coli. S. aureus* is one of the most common causative agents of mastitis and can cause both clinical and non-clinical forms of mastitis (2). *S. aureus* can cause a variety of infectious diseases in humans and animals by producing a variety of exotoxins and enzymes. Although *S. aureus* is a zoonotic pathogen, it can be transmitted to humans (3). *S. aureus* can be spread through milking tools, as well as through feces, urine, and other contaminants, and once it has infected a host, it is very difficult to eradicate (4). Mastitis is the most common and costly disease among dairy herds worldwide. Currently, *S. aureus*-induced mastitis is one of the biggest problems the dairy industry faces, with strong negative impacts on productivity, food safety, and public health. Therefore, there is an urgent need to find effective methods for prevention and control (5).

*Pueraria lobata* is a medicinal herb that has been used for the treatment of many diseases, such as cardiovascular disorders and diabetes. Puerarin is the main isoflavonoid active ingredient in *P. lobata* and has a wide range of pharmacological effects (6). A previous study revealed that puerarin has the potential to treat conditions such as osteoarthritis and cardiovascular disease (7). Moreover, puerarin can cross the blood-brain barrier and exert neuroprotective effects (8). Puerarin can alleviate various types of stimulus-induced organelle damage, including damage to the mitochondria, endoplasmic reticulum, and nucleus. In addition, puerarin has been shown to significantly improve the treatment of chronic metabolic diseases such as type two diabetes mellitus, pathological cardiac remodeling, and brain disease (9). A recent study showed that puerarin had no influence on the growth of *S. aureus* (10). Puerarin was also found to have a therapeutic effect on *S. aureus*-induced endometritis in mice by inhibiting ferroptosis (11). However, the therapeutic effect of puerarin against *S. aureus*-induced mastitis has not been thoroughly investigated.

Ferroptosis is a form of cell death characterized by lethal iron-dependent lipid peroxidation. The typical morphology of small mitochondria includes condensed mitochondrial membrane density, a reduction in or disappearance of mitochondrial cristae, and outer mitochondrial membrane rupture (12). Biochemically, ferroptosis is characterized by chemical or mutational inhibition of the cystine/glutamate antiporter, culminating in the accumulation of reactive oxygen species (ROS) in the form of lipid hydroperoxides (13). Like other types of apoptotic or non-apoptotic cell death, ferroptosis is regulated by functional genes or proteins, such as glutathione peroxidase 4 (GPX4), solute carrier family 7 member 11 (SLC7A11), and nuclear factor erythroid 2-like (14). Furthermore, in mastitis, inflammation is accompanied by the activation of phospholipases, which produce arachidonic acid (AA) from membrane phospholipids (15). AA metabolites are required for the cyclooxygenase and lipoxygenase (LOX) enzyme systems, and AA is converted through three metabolic pathways (16). Metabolites are converted to precursors of biologically active pro-inflammatory mediators, which are central to the pathways of inflammation (17). These features of mastitis are highly consistent with the features of iron toxicity.

The aim of this work was to study the therapeutic effect of puerarin on *S. aureus*-induced mastitis and to provide a theoretical basis for the clinical use of puerarin against mastitis caused by *S. aureus* infection.

## RESULTS

### The effects of puerarin on *S. aureus*-induced mammary histopathologic injury

**Fig 1:**
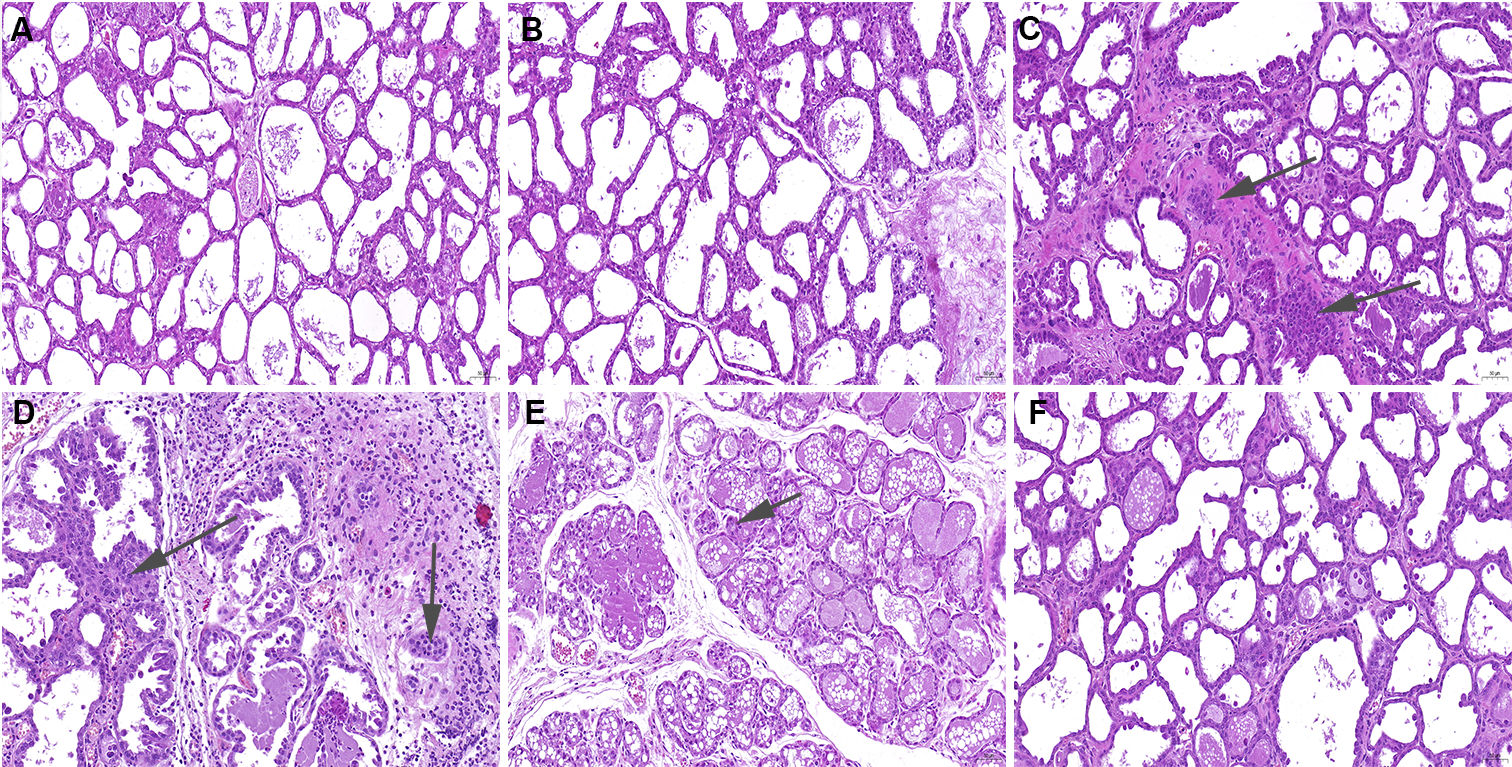
Effects of puerarin on *S. aureus*-induced mammary histopathological changes. Histopathologic sections of mammary tissues (H&E, ×100). Twenty-four hours after puerarin treatment, the mammary gland tissues were collected for the histological analysis by H&E staining (**A**) control, (**B**) puerarin (100 mg/kg) group, (**C**) *S. aureus* group, and (**D–F**) Puerarin (25, 50, and 100 mg/kg) + *S. aureus* groups.

To detect the protective effect of puerarin, mammary gland pathological injury was detected by hematoxylin and eosin (H&E) staining. Under a light microscope, the mammary gland follicles in the blank control group (Fig. 1A) and puerarin (100 mg/kg) group (Fig. 1B) were intact; no inflammatory cells were observed in the follicles, and no obvious pathological histological changes were observed. Compared with those in the control group, the mammary follicular alveoli in the *S. aureus* group were destroyed and infiltrated by many inflammatory cells (Fig. 1C). Histologically, puerarin (25, 50, and 100 mg/kg) treatment markedly attenuated *S. aureus*-induced mammary gland damage, such as edema and inflammatory cell infiltration (Fig. 1D through F).

### Puerarin inhibits *S. aureus*-induced MPO activity

**Fig 2:**
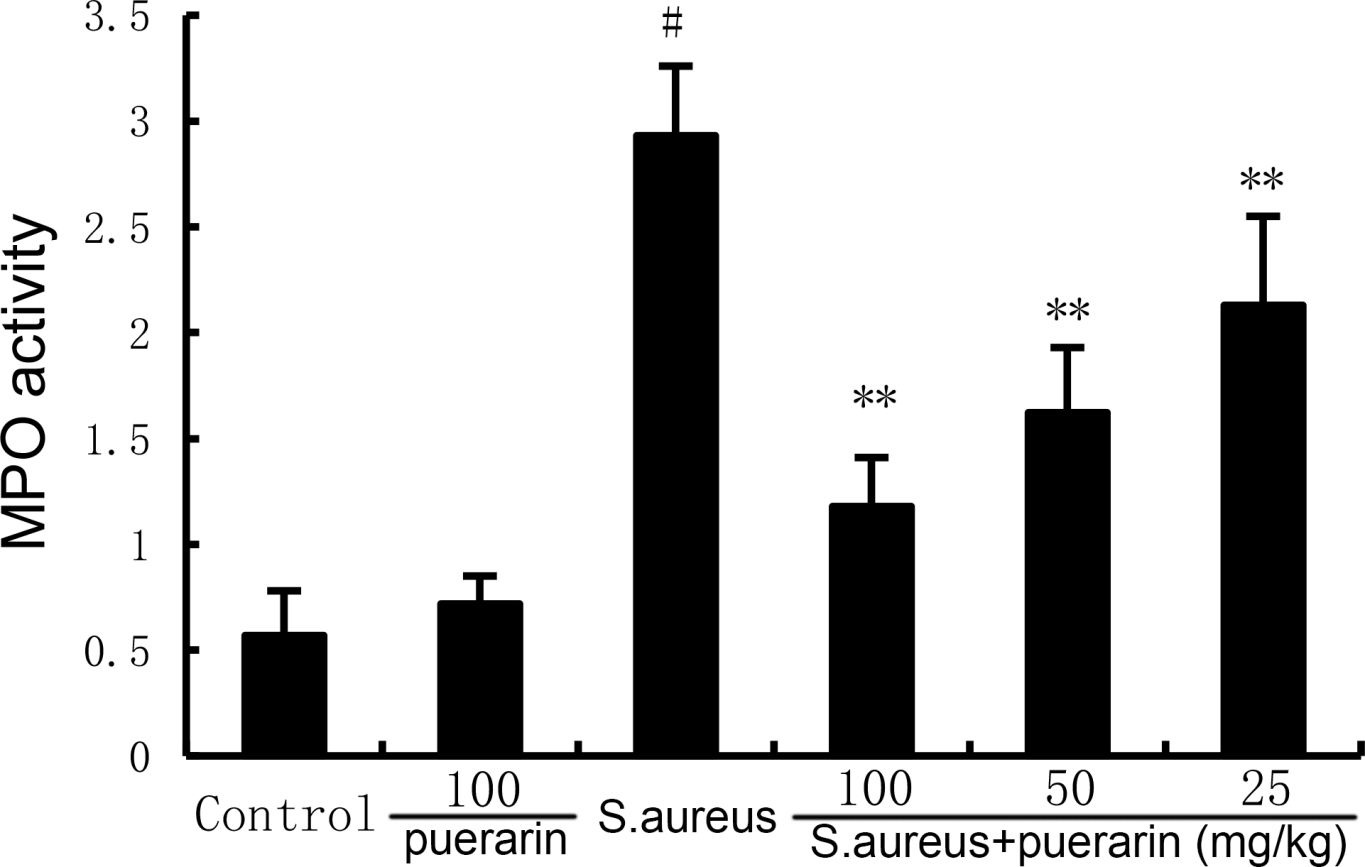
Effect of puerarin on MPO activity in the mammary gland. Twenty-four hours after puerarin treatment, the mammary gland tissues were collected, and the MPO activity in mammary gland tissues was detected by the MPO detection kit. The values presented are the mean ± SD and were analyzed using one-way ANOVA. ^#^*P* < 0.01 indicates a significant difference from the control group; **P < 0.01 indicates a significant difference from the *S. aureus* group.

The myeloperoxidase (MPO) activity in the mammary gland tissue of the mice was measured using a biochemical reagent kit. *S. aureus* treatment resulted in increased levels of MPO activity in mammary tissue compared to the normal control group. In contrast, puerarin treatment attenuated *S. aureus*-induced mammary MPO activity (Fig. 2).

### Puerarin alleviates *S. aureus*-induced TNF-α and IL-1β production

**Fig 3:**
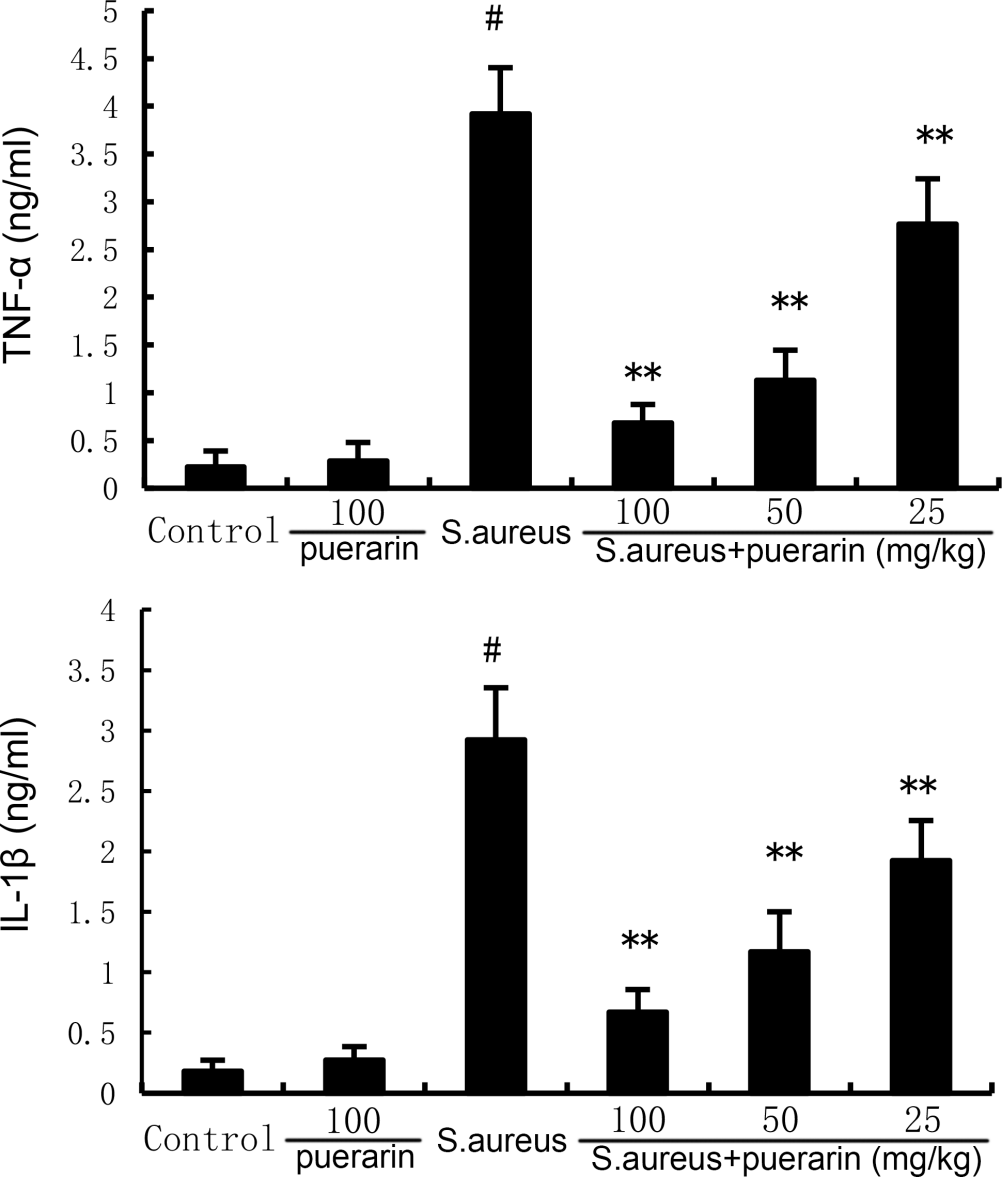
Effect of puerarin on inflammatory cytokine production in the mammary gland. Twenty-four hours after puerarin treatment, the mammary gland tissues were collected, and the levels of inflammatory cytokines were measured by ELISA. The values presented are the mean ± SD and were analyzed using one-way ANOVA. ^#^*P* < 0.01 indicates a significant difference from the control group; **P < 0.01 indicates a significant difference from the *S. aureus* group.

The levels of tumor necrosis factor-α (TNF-α) and interleukin-1β (IL-1β) in mammary glands were measured by enzyme-linked immunosorbent assay (ELISA). *S. aureus* infection resulted in increased TNF-α and IL-1β levels in mammary tissues, which indicated increased systemic inflammation in the mice. However, puerarin significantly reduced the *S. aureus*-induced increase in TNF-α and IL-1β levels in mammary tissues. These results suggest that puerarin attenuates the systemic inflammatory response in mammary tissues (Fig. 3).

### Puerarin suppresses ferroptosis in the mammary tissue

**Fig 4:**
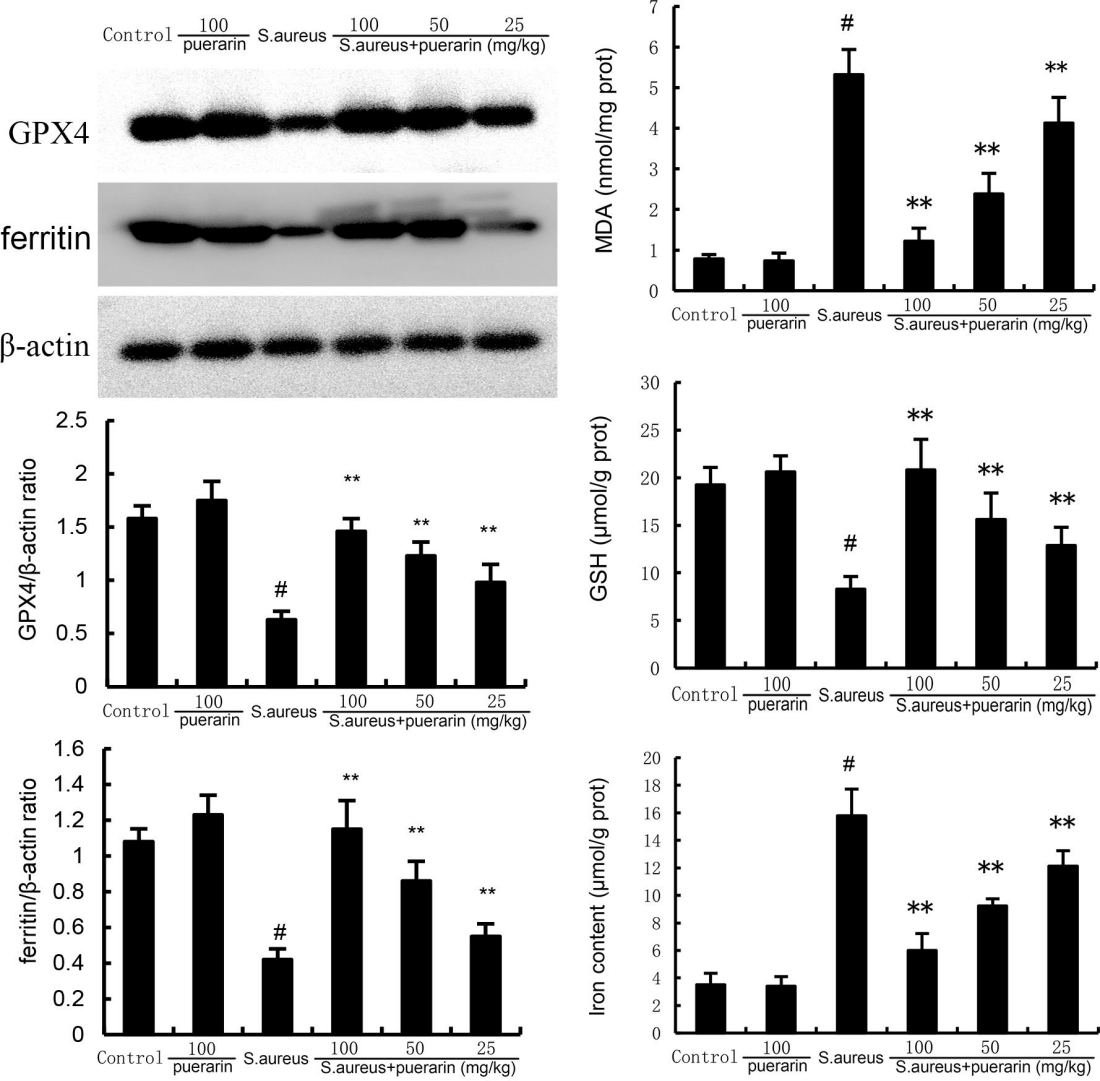
Effect of puerarin on GPX4 and ferritin expression, MDA, iron, and GSH production in the mammary gland. Twenty-four hours after puerarin treatment, the mammary gland tissues were collected, and the MDA, iron, and GSH production in mammary gland tissues were detected by the detection kits. The expression of GPX4 and ferritin was detected by western blot analysis. The values presented are the mean ± SD and were analyzed using one-way ANOVA. ^#^*P* < 0.01 indicates a significant difference from the control group; **P < 0.01 indicates a significant difference from the *S. aureus* group.

To investigate the effects of puerarin on ferroptosis in *S. aureus*-induced mastitis, we first determined ferritin and GPX4 expression in *S. aureus*-induced mastitis. We found that ferritin and GPX4 expression was decreased in *S. aureus*-infected mice. Puerarin treatment significantly increased ferritin and GPX4 expression. Ferroptosis is characterized by iron accumulation, lipid peroxidation, and damage to the antioxidant system. Therefore, we examined the mammary iron content, and MDA and GSH levels. As expected, *S. aureus* infection increased the levels of Fe^2+^ and MDA but decreased the relative expression of GSH. However, these changes were reversed by puerarin treatment (Fig. 4). These results suggest that puerarin can inhibit *S. aureus*-induced ferroptosis.

### Puerarin inhibits *S. aureus*-induced NF-κB activation

**Fig 5:**
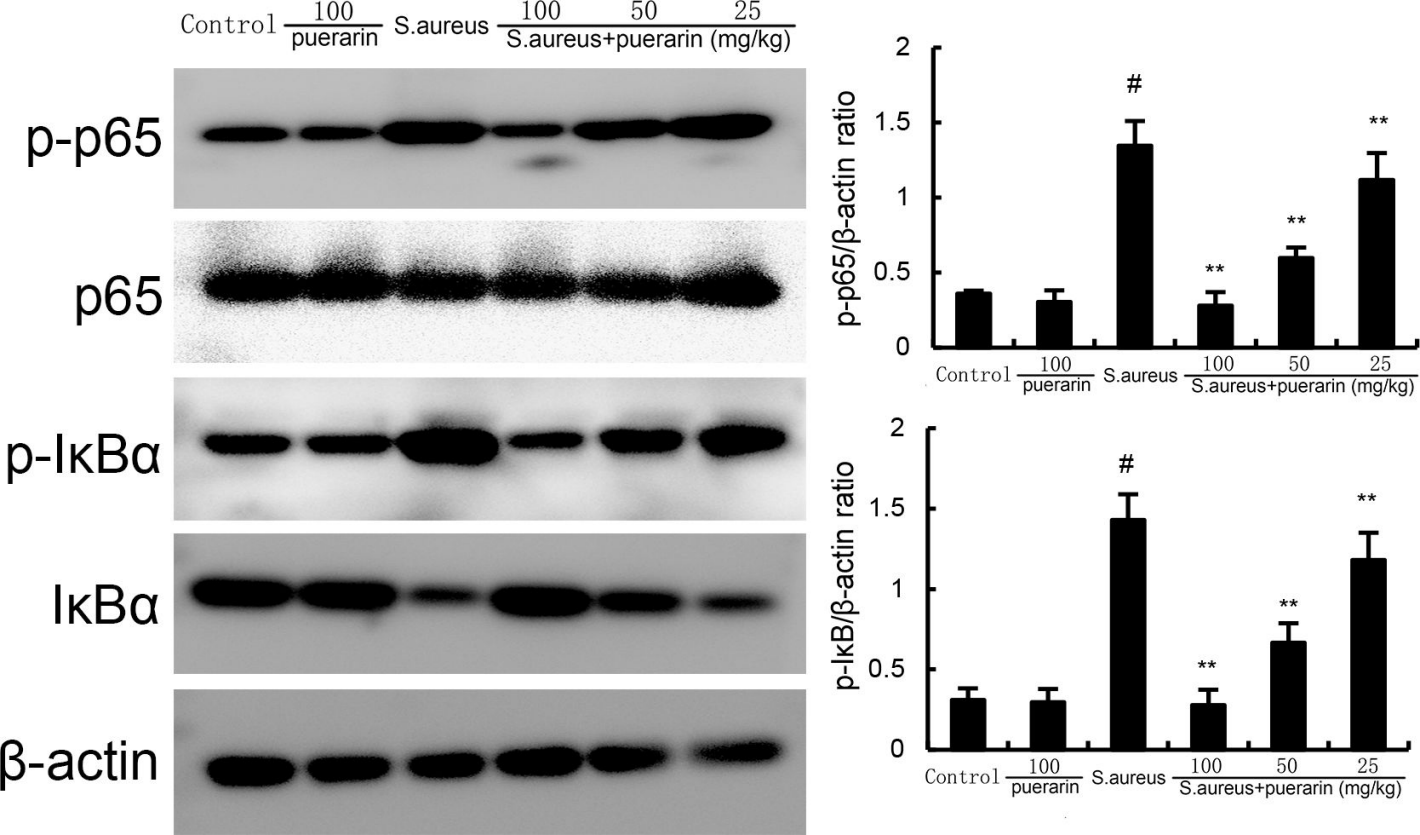
Effect of puerarin on NF-κB activation in the mammary gland. Twenty-four hours after puerarin treatment, the mammary gland tissues were collected, and the expression of NF-κB signaling pathway proteins was measured by western blot analysis. The values presented are the mean ± SD and were analyzed using one-way ANOVA. ^#^*P* < 0.01 indicates a significant difference from the control group; **P < 0.01 indicates a significant difference from the *S. aureus* group.

To investigate the anti-inflammatory mechanism of puerarin in *S. aureus*-induced mastitis, we first determined the levels of phosphorylated IκB and NF-κB p65 in *S. aureus*-induced mastitis. We found that phosphorylated inhibitor of nuclear factor kappa-B (IκB) and nuclear factor kappa-B (NF-κB) p65 expression increased in *S. aureus*-infected mice. Puerarin treatment attenuated NF-κB activation in *S. aureus*-treated mice (Fig. 5).

### Effects of puerarin on the SIRT1/p53/SLC7A11 signaling pathway

**Fig 6:**
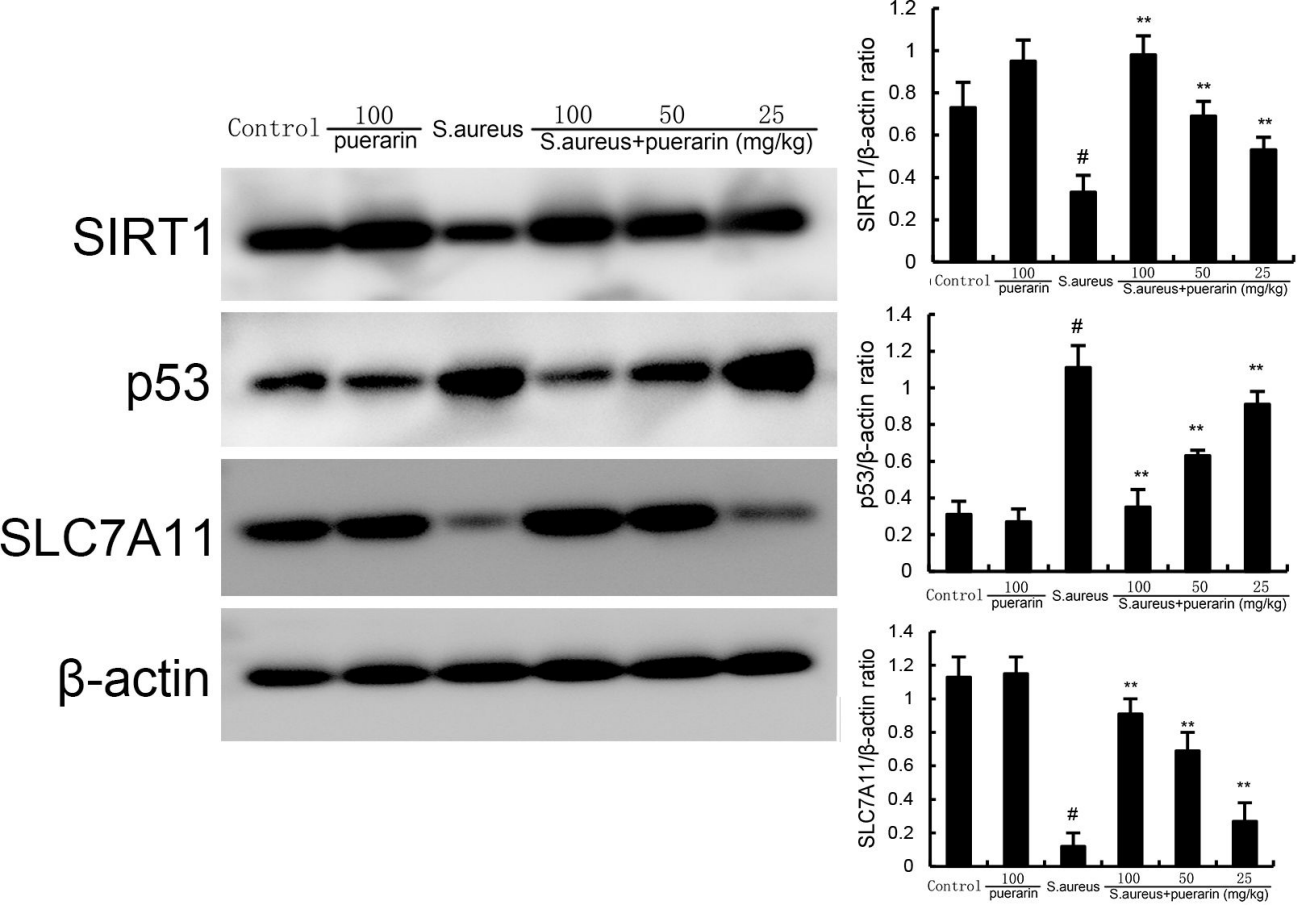
Effect of puerarin on SIRT1, p53, and SLC7A11 expression in the mammary gland. Twenty-four hours after puerarin treatment, the mammary gland tissues were collected, and the expression of SIRT1, p53, and SLC7A11 was measured by western blot analysis. The values presented are the mean ± SD and were analyzed using one-way ANOVA. ^#^*P* < 0.01 indicates a significant difference from the control group; **P < 0.01 indicates a significant difference from the *S. aureus* group.

To further explore the mechanism of the anti-ferroptotic effect of puerarin, we examined the protein expression of SIRT1, p53, and SLC7A11. The protein expression of SIRT1 and SLC7A11 decreased upon *S. aureus* infection, and this effect was significantly antagonized by puerarin treatment. Moreover, p53 expression increased upon *S. aureus* infection, and this effect was significantly antagonized by puerarin treatment (Fig. 6).

### SIRT1 inhibitor reverses the inhibitory effects of puerarin on *S. aureus*-induced inflammation and ferroptosis

**Fig 7:**
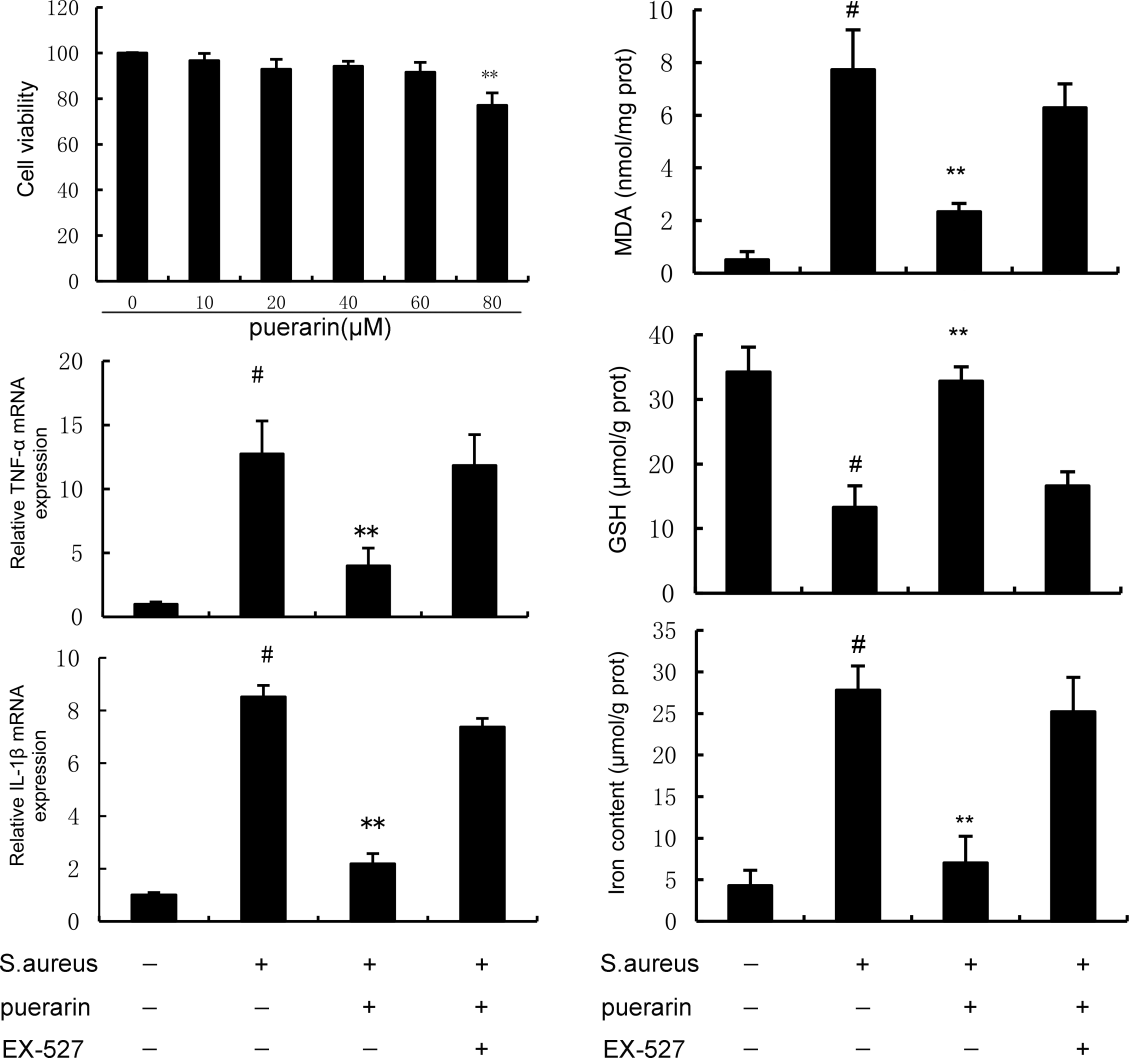
SIRT1 inhibitor reverses the inhibition of puerarin on *S. aureus*-induced inflammation and ferroptosis. The effect of puerarin on the viability of mMECs was detected by the CCK8 method. mMECs were treated with EX-527 (10 µm) and puerarin (60 µm) and stimulated with *S. aureus*. Samples were collected after 24 hours for follow-up testing. The expression of IL-1β and TNF-α was detected by qRT-PCR. The production of ferroptosis indicators (MDA, GSH, and Fe^2+^) was detected by the detection kits. The values presented are the mean ± SD and were analyzed using one-way ANOVA. ^#^*P* < 0.01 indicates a significant difference from the control group; **P < 0.01 indicates a significant difference from the *S. aureus* group.

To further confirm that the SIRT1/p53/SLC7A11 signaling pathway is involved in the anti-inflammatory and anti-ferroptotic effects of puerarin, the SIRT1 inhibitor EX-527 was added to the *in vitro* experiments. The Cell Counting Kit-8‌ (CCK8) data revealed that puerarin at doses ranging from 0 to 60 μm did not affect the viability of mouse mammary epithelial cells (mMECs) (Fig. 7). Moreover, the inhibitory effects of puerarin on *S. aureus*-induced inflammation and ferroptosis were prevented by SIRT1 inhibitor EX-527 (Fig. 7).

## DISCUSSION

*S. aureus* is one of the major pathogens causing mastitis in dairy ruminants. The establishment of relevant treatments for *S. aureus*-induced mastitis is essential to avoid economic losses in the dairy industry. Puerarin is the main active isoflavonoid ingredient in *P. lobata* and has been reported to have anti-inflammatory and anti-oxidative properties. However, there are currently few reports on the therapeutic effect of puerarin on mastitis. In this study, we investigated the therapeutic effect of puerarin on *S. aureus*-induced mastitis.

Mastitis is a common and serious disease. The systemic inflammatory response in dairy ruminants involves the activation of inflammatory pathways such as NF-κB, leading to the expression of many genes, such as those encoding inflammatory cytokines (18). Increased inflammatory cytokines were observed in mastitis. Many studies have confirmed that the inhibition of these inflammatory cytokines can attenuate mammary histopathologic injury (19). In this study, puerarin inhibited the up-regulation of the pro-inflammatory factors TNF-α and IL-1β in mammary tissues infected with *S. aureus*. Western blot assays revealed that puerarin could reduce the phosphorylation of NF-κB/p65 induced by *S. aureus*. Our results suggest that puerarin can inhibit the activation of the NF-κB pathway and the expression of pro-inflammatory factors, thereby reducing inflammation induced by *S. aureus*.

Ferroptosis is a form of programmed cell death that is largely dependent on iron ions (12). At the onset of mastitis, the inflammatory response may lead to elevated intracellular levels of iron ions, which induce the onset of ferroptosis. In addition, inflammatory cells play an important role in mastitis, and they can also take up large amounts of iron ions and secrete large amounts of ROS, exacerbating cellular ferroptosis. Ferroptosis is closely linked with oxidative stress (20). Cellular oxidative stress involves a variety of signaling pathways and has important roles in developmental processes, normal physiological activities, and a variety of pathological processes. Oxidative stress plays an important role in promoting ferroptosis (21). On the one hand, oxidative stress can lead to the release of intracellular iron, increasing its concentration and promoting ferroptosis. On the other hand, oxidative stress affects the balance of other intracellular metabolic pathways related to ferroptosis, thus further regulating ferroptosis. In this study, puerarin treatment reduced ferroptosis-related pathway activity and oxidative stress levels in mammary tissues. We examined the activity of the antioxidant enzyme GSH and found that *S. aureus* reduced GSH activity. However, puerarin treatment prevented the *S. aureus*-induced reduction in antioxidants. *S. aureus* greatly increased the level of the lipid peroxidation product enzyme MDA, whereas puerarin reversed this effect, suggesting that puerarin has a protective effect against *S. aureus*-induced oxidative stress. Moreover, the ferroptosis-related proteins GPX4 and ferritin were markedly up-regulated by puerarin. These data indicate that puerarin can suppress *S. aureus*-induced ferroptosis.

SIRT1 is an NAD^+^-dependent deacetylase that plays a role in maintaining mitochondrial function and regulating oxidative stress (22). SLC7A11, a cysteine/glutamic acid reverse transporter protein, participates in the transport of amino acids through the plasma membrane and regulates the mechanism of ferroptosis (23). In recent years, an increasing number of studies have shown that SLC7A11 is closely related to the occurrence and development of many diseases (24). Recent studies revealed that activating the SIRT1/p53/SLC7A11 signaling pathway protects against sepsis-induced cardiomyopathy through ferroptosis suppression (25). Additionally, activating the SIRT1/p53/SLC7A11 signaling pathway inhibits lung inflammation and injury by attenuating ferroptosis (26). In this study, puerarin activated the SIRT1/p53/SLC7A11 signaling pathway, suggesting that puerarin inhibited ferroptosis through the SIRT1/p53/SLC7A11 signaling pathway. In future research, we will further determine the mechanism of puerarin-dependent attenuation of mastitis, for example, through experiments utilizing gene-deficient mice. Furthermore, the therapeutic effect of puerarin on mastitis induced by other pathogens will also be explored. The effect of puerarin on mastitis is achieved mainly through the inhibition of inflammation, oxidative stress, and ferroptosis. The function of puerarin is to alleviate the symptoms of mastitis, not to prevent infection. Whether puerarin can treat mastitis in clinical practice still requires many experiments to confirm, such as experiments on cows. This study revealed that only puerarin has the potential to treat mastitis and that it cannot replace antibiotics to treat mastitis.

In conclusion, puerarin can alleviate the inflammatory damage to mammary tissues induced by *S. aureus* and exert anti-inflammatory and anti-ferroptotic effects. The mechanism may be through the activation of the SIRT1/p53/SLC7A11 signaling pathway.

## MATERIALS AND METHODS

### Reagent

Puerarin was obtained from Sigma-Aldrich. The antibodies used in this study were obtained from CST (Boston, USA). The MPO, MDA, and GSH detection kits were purchased from Nanjing Jiancheng Institute of Bioengineering (Nanjing, China). ELISA kits for the measurement of TNF-α and IL-1β levels were purchased from BioLegend (California, USA).

### Animals

A total of 72 adult postpartum and lactating Bagg Albino c mice (8–10 weeks) were used in this study. These mice were purchased from the Experimental Animal Center of Baiqiuem Medical College, Jilin University (China). The mice were raised in a temperature-controlled room (23 ± 21°C) with a standardized light/dark cycle (12/12 hours) and free access to food and water.

### Experimental design and grouping

Seventy-two mice were randomly divided into six groups of 12 mice in each group: the control group, puerarin (100 mg/kg) group, *S. aureus* group, and *S. aureus *+ puerarin (25, 50, and 100 mg/kg) groups. The mouse model of *S. aureus*-induced mastitis was generated as follows: 100 µL of *S. aureus* (containing 10^8^ colony-forming unit bacteria) suspension was injected into both mammary glands (R4 and L4) of pentobarbital-anesthetized mice. Twenty-four hours after infection, puerarin (25, 50, and 100 mg/kg) was injected intraperitoneally. The control mice were given equal amounts of phosphate buffered saline. At the end of the treatment, all the mice were killed, and the mammary glands were collected and stored at −80°C until analysis.

### *In vitro* experiment

mMECs were obtained from American Type Culture Collection (ATCC CRL-3063). The mMECs were cultured in Dulbecco′s modified eagle medium containing 10% fetal bovine serum at 37°C in a 5% CO₂ incubator. After being passaged to the logarithmic growth stage, the cells were seeded in a 6-well plate at a density of 1 × 10⁵ cells/mL. The infection test was conducted when the degree of cell fusion reached 80% to 90%. The effect of puerarin on the viability of mMECs was detected with the CCK8 method. mMECs were treated with EX-527 (10 µm) or puerarin (60 µm) and stimulated with *S. aureus*. EX-527 was dissolved in dimethyl sulfoxide‌ (DMSO), and the cells in the control group were treated with an equal amount of DMSO. Samples were collected after 24 hours for follow-up testing. The expression levels of IL-1β and TNF-α were detected by qRT-PCR. Ferroptosis indicators (MDA, GSH, and Fe^2+^) were detected with the detection kits.

### H&E staining

Mammary tissue samples used for the histological analysis were fixed in 4% paraformaldehyde for 48 hours and embedded in paraffin to prepare 5 µm paraffin sections. Then, the samples were dewaxed with xylene twice and hydrated with ethanol. Furthermore, all hydrated sections were stained with H&E and examined under an optical microscope (Olympus, Tokyo, Japan).

### MPO and ferroptosis assays

MPO activity in mammary gland tissues was detected using the MPO kit according to the manufacturer’s instructions. The absorbance at a wavelength of 460 nm was recorded, and the enzyme activity was determined. Ferroptosis indicators (MDA, GSH, and Fe^2+^) in each group were measured with detection kits, and all the steps were performed in strict accordance with the instructions of the detection kits.

### Inflammatory cytokine assay

The levels of pro-inflammatory cytokines (IL-1β and TNF-α) in each group were measured by ELISA, and all the steps were performed in strict accordance with the ELISA kit instructions.

### Western blot analysis

The total protein of the mammary gland was harvested and extracted using a tissue protein extraction kit (Thermo Fisher Scientific, USA). SDS-PAGE (10%) was used to separate the proteins, which were then transferred to polyvinylidene fluoride membranes. The membrane was blocked with 5% fat-free milk for 2 hours at room temperature and incubated overnight at 4°C with primary antibodies. After 15 minutes with three washes with tris buffered saline with tween (0.05% tween 20), the membrane was incubated with goat anti-rabbit horseradish peroxidase (1:5,000; CST, USA) for 2 hours at room temperature, and then washed again. An Immobilon Western Chemiluminescent HRP Substrate (ECL) (Millipore, USA) was used for visualization. All western blots were analyzed using Image-Pro Plus 6.0 (Media Cybernetics). β-actin was used as an internal reference protein.

### Statistical analysis

GraphPad Prism version 8.0 (San Diego, CA, USA) was used for all the analyses, and all the data are expressed as the means ± SD. One-way analysis of variance (ANOVA) was used for the comparisons between more than two groups, followed by Tukey’s test.

## Data Availability

The data that support the findings of this study are available from the corresponding author upon reasonable request.
